# Quasispecies Theory and the Behavior of RNA Viruses

**DOI:** 10.1371/journal.ppat.1001005

**Published:** 2010-07-22

**Authors:** Adam S. Lauring, Raul Andino

**Affiliations:** 1 Department of Medicine, University of California, San Francisco, San Francisco, California, United States of America; 2 Department of Microbiology and Immunology, University of California, San Francisco, San Francisco, California, United States of America; University of California San Diego, United States of America

## Abstract

A large number of medically important viruses, including HIV, hepatitis C virus, and influenza, have RNA genomes. These viruses replicate with extremely high mutation rates and exhibit significant genetic diversity. This diversity allows a viral population to rapidly adapt to dynamic environments and evolve resistance to vaccines and antiviral drugs. For the last 30 years, quasispecies theory has provided a population-based framework for understanding RNA viral evolution. A quasispecies is a cloud of diverse variants that are genetically linked through mutation, interact cooperatively on a functional level, and collectively contribute to the characteristics of the population. Many predictions of quasispecies theory run counter to traditional views of microbial behavior and evolution and have profound implications for our understanding of viral disease. Here, we discuss basic principles of quasispecies theory and describe its relevance for our understanding of viral fitness, virulence, and antiviral therapeutic strategy.

## Introduction

The rapid evolution of RNA viruses complicates the management of chronic infections and the control of emerging infectious agents [1]–[3]. The ongoing global AIDS pandemic and the resurgence of influenza highlight the difficulties associated with these genetically labile pathogens [4]–[6]. RNA viruses have also been responsible for recent sporadic epidemics of emerging and reemerging viral diseases including dengue, West Nile fever, and Ebola [7], [8]. Because of their high mutation rates, these viruses are moving targets for therapeutic intervention and frequently develop resistance to vaccines and antiviral drugs [9]. A clearer understanding of viral evolutionary dynamics and its relationship to virulence and drug resistance may facilitate the development of more effective therapeutics.

The evolutionary dynamics of RNA viruses are complex and their high mutation rates, rapid replication kinetics, and large population sizes present a challenge to traditional population genetics [10]. Quasispecies theory is a mathematical framework that was initially formulated to explain the evolution of life in the “precellular RNA world [11].” It builds on classical population genetics, but seeks to explore the consequences of error-prone replication and near-infinite population sizes for genome evolution [12], [13]. More recently, quasispecies theory has been used to describe the evolutionary dynamics of RNA viruses, and many of its predictions have been validated experimentally in model systems [2], [14], [15]. Some of these observations challenge more traditional views of evolution and have profound implications for the control and treatment of viral diseases.

Here we explain basic aspects of quasispecies theory, describe key experiments that define “quasispecies effects,” and highlight how these results may shape our view of viral pathogenesis, antiviral drug development, and vaccine design. We will stress three clinically relevant principles. First, the fitness of a particular virus sequence may be determined more by its freedom to mutate into related sequences than by its own replicative capacity. Second, many viruses operate near a threshold of “error catastrophe” and may be combated by increasing their replication error rates. Third, increasing the fidelity of genome replication may paradoxically attenuate viruses.

## Error-Prone Replication and Viral Quasispecies

Most viruses encode enzymes responsible for replicating their DNA or RNA genomes. The intrinsic error rate, or fidelity, of the replicase determines the mutation rate for that virus and the range of genetic variation upon which natural selection can act. Viral RNA polymerases exhibit characteristically low fidelity with measured mutation rates of roughly 10^−4^ mutations per nucleotide copied, which is orders of magnitude greater than those of nearly all DNA-based viruses and organisms [10], [15], [16]. Given the large population sizes observed in both experimental and natural infections, it is estimated that every possible point mutation and many double mutations are generated with each viral replication cycle and may be present within the population at any time [17]. Because RNA viruses exist as swarms of similar variants that are continuously regenerated by mutation of related sequences, our ability to predict the outcome of an infection or a therapeutic intervention from studies of isolated clones is limited. Even a defined molecular clone will quickly transform into a collection of related sequences when introduced into cells. This collection is the quasispecies and is organized around a master sequence [12].

**Figure 1:**
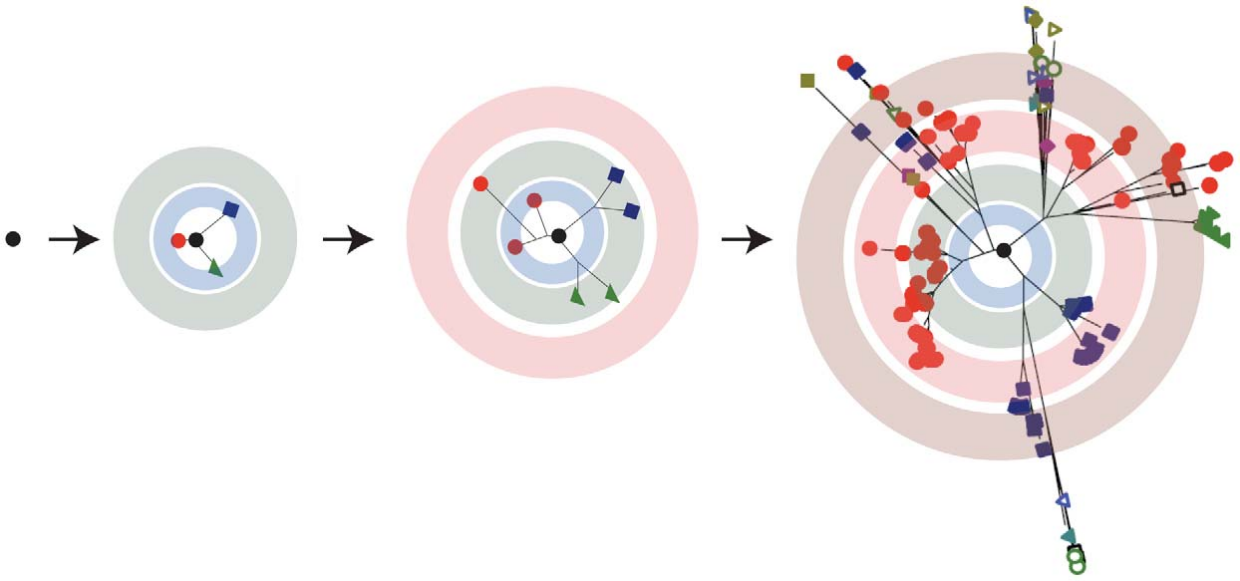
RNA viruses exist as a quasispecies. A virus replicating with a high mutation rate will generate a diverse mutant repertoire over the course of a few generations. In these trees, each branch indicates two variants linked by a point mutation and the concentric circles represent serial replication cycles. The resulting distribution is often represented as a cloud centered on a master sequence. This two dimensional schematic is a vast oversimplification of the intraquasispecies connectivity. In the mathematical formulations of quasispecies theory, sequence space is multidimensional, with numerous branches between variants.

The genetic organization of populations is often depicted using the concept of sequence space, a geometric representation of all possible sequences where physical distance reflects genetic similarity. For example, a given viral genome will undergo replication and generate hundreds of progeny that differ at roughly one position (Figure 1). Subsequent rounds of replication will generate a more complex mutant distribution with variants lying farther away from each other in sequence space. This ensemble of mutants forms a “cloud” of variants, or quasispecies, in which mutation generates a swarm of candidate genomes that is pruned by natural selection. According to population genetics, the frequency of a given variant in a population is closely approximated by its ability to survive and reproduce—its fitness. In quasispecies formulations, where mutation rates are elevated, frequency is also subject to the probability that the variant will be generated de novo by mutation of its neighbors in sequence space [12]. In RNA viruses, the contribution of mutation to genotype frequency is significant, and variants are “coupled” in sequence space [18]. That is, a low fitness variant can be maintained at a higher than expected frequency because it is coupled to a well-represented, higher fitness genotype in sequence space. The phenomenon of mutational coupling is one of the defining characteristics of a quasispecies, as it places individual mutants within a functional network of variants [2].

**Figure 2:**
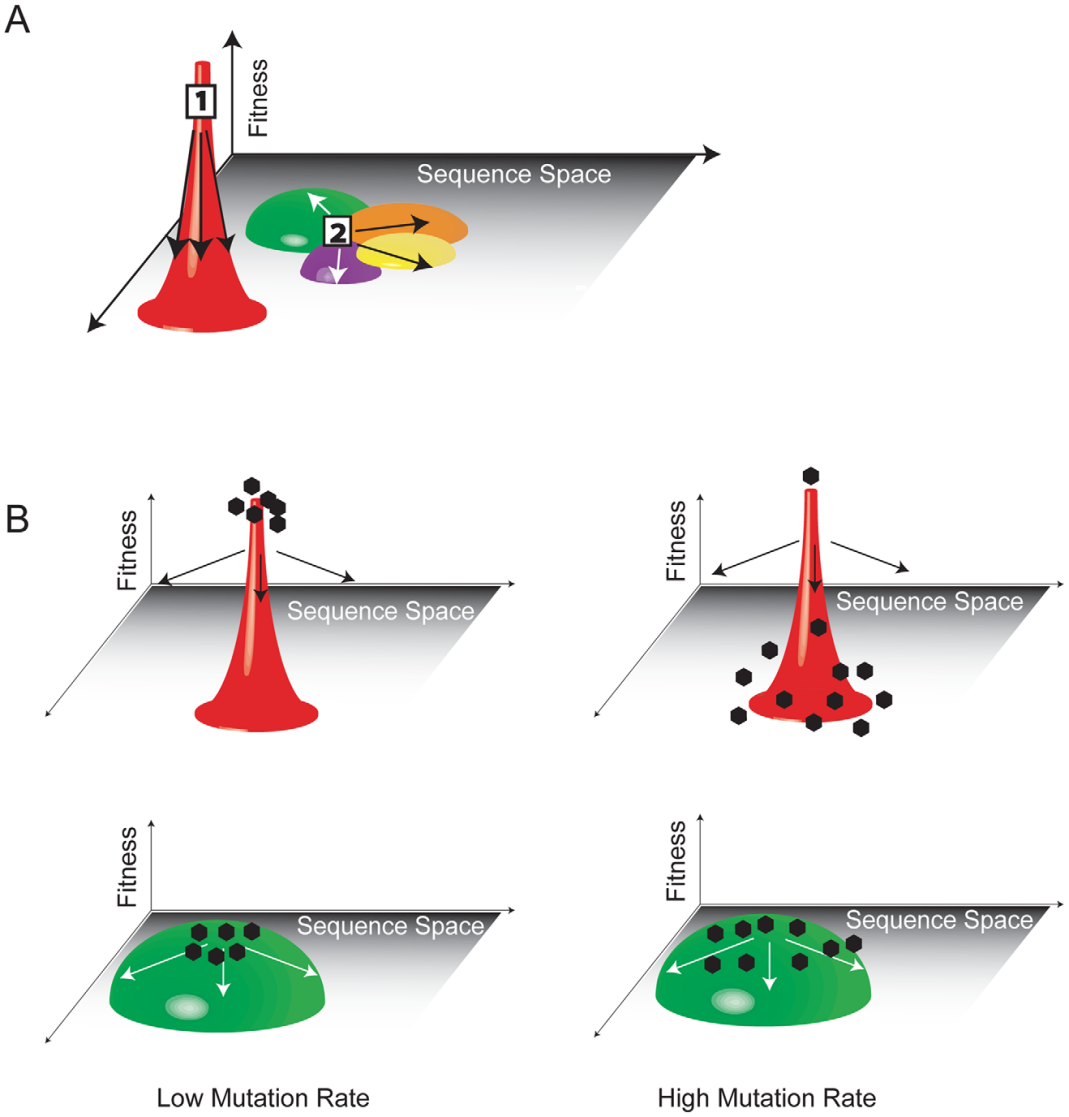
The fitness landscape and survival of the flattest. (A) Population 1 has high fitness but is trapped in sequence space because mutation leads to a dramatic loss of fitness. Population 2 is more mutationally robust because mutation leads to minor fitness losses. The flatter population is ideally situated to move through sequence and access other local peaks through neighboring mutational networks (indicated in different colors). (B) At low mutation rates, variants will be genotypically stable and cluster at the top of the fitness peak. The variant with the highest fitness will easily outcompete all others. At high mutation rates, variants spread out over the corresponding peaks. Variants on the flatter peak (green) remain near their fitness optimum and have a higher mean fitness than the population located on the steeper peak (red). The flatter population will prevail.

Viral populations evolve within a fitness landscape where the “ground level” is a representation of the range of genotypes in sequence space. The “altitude” at any given location is the fitness associated with that particular genotype. The environment and its selective pressures determine the contours of the corresponding landscape, and adaptation to an environment involves a mutational walk from one point in the fitness landscape to another (Figure 2A). In quasispecies theory, a network of mutationally coupled variants will span the corresponding peaks and valleys of the fitness landscape. A fast replicating population well suited to a given environment will inhabit a high and narrow peak in the fitness landscape, while a less fit but more genetically diverse population will occupy a lower, broader one.

A viral quasispecies, then, is a cloud of diverse variants that are genetically linked through mutation, interact cooperatively on a functional level, and collectively contribute to the characteristics of the population. The unit of selection is the population as a whole, and the nature of the functional interactions among genetically distinct variants is therefore of critical importance to pathogenesis in infected hosts. These effects and their biomedical implications are described below.

## The Problem of Fitness and Survival of the Flattest

Mutation and selection are the most fundamental processes in evolution. In Darwinian evolution, natural selection acts on existing genetic variation to optimize fitness. Conceptually, fitness refers to how well a given organism “fits” into its environment, often reflected in how well it survives and reproduces [19]. In experimental settings, precise fitness measurements are paramount, and virologists typically use replicative capacity as a surrogate for fitness [20]. While replication is useful as a first approximation, other factors such as immune escape, transmissibility, and cellular tropism are important components of fitness in the dynamic host environment [21]. Furthermore, because quasispecies theory adds the complexity of mutant networks, we must incorporate a population-based model into our fitness definition.

Measuring the fitness of individual variants within a population may misrepresent the fitness of a quasispecies. Early experiments with vesicular stomatitis virus (VSV) established that high fitness variants could be suppressed to low levels within a complex population [22]. Conversely, longitudinal studies of dengue virus isolates have identified defective clones that are stably maintained at a high frequency in the population [23]. Further consideration of the fitness landscape model may explain these paradoxical results (Figure 2B). Consider two populations, generated from either a fast replicator or a slower one. At low mutation rates, the fast replicator will triumph because its progeny are genetically identical and generated more quickly. In the fitness landscape, this population occupies a tall, narrow peak, where there is little genotypic diversity and maximal fitness. In RNA viruses, elevated mutation rates mean that a fast replicator will give rise to genetically diverse progeny, many of which are significantly less fit than the parent. Quasispecies theory predicts that slower replicators will be favored if they give rise to progeny that are on average more fit; these populations occupy short, flat regions of the fitness landscape [18]. This effect, termed survival of the flattest, has been observed in digital organisms, bacteriophages, and VSV [24]–[28]. Flat quasispecies accept mutation without a corresponding effect on fitness, and these mutationally robust populations form a large, selectively neutral network of variants in sequence space. While neutral mutations do not change the corresponding phenotype, they may have epistatic effects on subsequent mutations and redraw the genotype–phenotype map [29].

A flat quasispecies with an expansive mutant repertoire can explore vast regions of sequence space without consequence and is poised to adapt to rapid environmental change. This framework may explain many observed phenomena with direct clinical relevance. Arboviruses must successfully adapt to insect and mammalian hosts and their associated fitness landscapes. A quasispecies that occupies a broad, flat region of sequence space could access neutral networks and local fitness peaks in either environment. Similarly, retrospective studies of primary HIV isolates suggest that HIV could be moving to a flatter and less fit region of sequence space [20], [30]–[32]. In the case of influenza, detailed antigenic mapping of the hemagglutinin protein suggests that interpandemic strains remain antigenically stable for years despite genetic drift and evolve over a neutral region of sequence space. This steady accumulation of genetic diversity is punctuated by epochal shifts in antigenicity [33]. Though this adaptive process occurs on the interhost level over a long time scale, it highlights the importance of “flatness” in viral evolution.

## Error Catastrophe

Given their high mutation rates, it is not surprising that many discussions of RNA virus evolution focus on the relationship between genetic diversity and adaptability. While it is clear that RNA viruses have the capacity to quickly explore large regions of sequence space, genome size and selective constraints place significant limitations on the amount of diversity that is actually expressed [34]. Most RNA virus genomes are relatively small and contain either overlapping reading frames or sequences that serve both coding and structural functions. Similarly, coding mutations that mediate escape from host immune surveillance may compromise protein function. In VSV, for example, approximately 60% of spontaneous mutations are deleterious [35]. Evolutionary theory would suggest that the short-term cost of mutation is balanced by the long-term benefit of adaptability in the face of a dynamic environment [36], [37].

**Figure 3:**
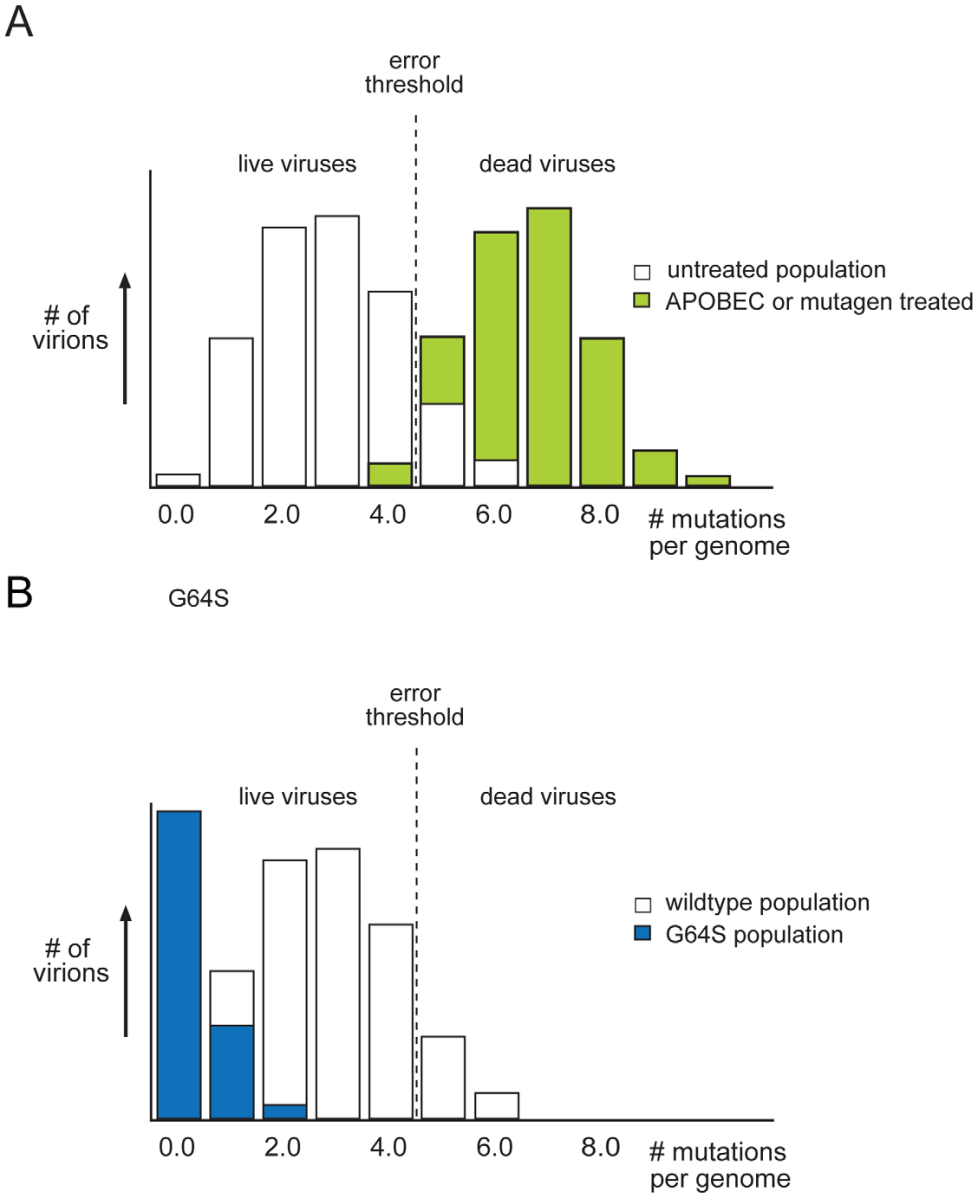
Mutant distributions and the error threshold. (A) The majority of viruses in a wild-type population has few mutations and is viable Some viruses, bearing a higher mutational load, are nonviable and considered beyond the threshold of error catastrophe (shown in green). Small increases in mutation frequency, mediated by host APOBEC proteins or exogenous mutagen, push the distribution to the right, past the error threshold. The number of errors per genome is sufficiently high to lethally mutate a majority of the population. (B) A high fidelity polymerase results results in a narrower quasispecies situated farther from the error threshold. This population is more resistant to the effect of mutagen, because it does not accumulate as many mutations, as the wild type does not cross the error threshold. Figure adapted from Crotty et al. [42].

Quasispecies theory accepts this trade-off, and proposes an upper limit to mutation rate, the “error threshold” (Figure 3A). According to Eigen's original formulations, a quasispecies can remain at equilibrium despite a high mutation rate [38], [39]. Small increases in mutation rate will upset this balance as the master sequence itself disappears and meaningful genetic information is lost in an avalanche of errors. Because Eigen compared this process to chemical phase transitions, it has been termed “mutational meltdown.” It is now clear that many RNA viruses replicate near the error threshold. Early studies with VSV showed that chemical mutagens generally reduced viral infectivity, and studies with poliovirus clearly demonstrated that mutagenic nucleoside analogs push viral populations to extinction [40]–[43]. The effect is dramatic—a 4-fold increase in mutation rate resulted in a 95% reduction in viral titer. Others have found similar results with lymphocytic choriomeningitis virus (LCMV) and foot and mouth disease virus (FMDV) [44]–[48]. The lethal mutagenesis of viral populations is perhaps the most important implication of the error threshold.

Recent work on host cell restriction of retroviral infection suggests that lethal mutagenesis is a natural form of antiviral defense [49]. The cytidine deaminase APOBEC3G was initially identified as a protein targeted by the HIV Vif protein during replication [50]. Humans have 11 APOBEC proteins that edit cellular messages by converting cytosine bases to uracil. Subsequent work demonstrated that APOBEC proteins could induce lethal mutagenesis of HIV through widespread deamination of the HIV genome during reverse transcription, and highly mutagenized HIV genomes with signatures of deamination have been observed in patients [51]–[56]. This mechanism appears to be evolutionarily conserved and active against hepatitis B virus, simple retroviruses, and endogenous retroelements [57]. While mutation-independent activities have also been identified, it is clear that APOBEC-mediated lethal mutagenesis is a critical cellular defense against RNA viruses. The fact that these pathogens replicate close to the error threshold makes them particularly sensitive to slight increases in mutational load (Figure 3A).

These observations suggest that lethal mutagenesis could be an effective therapeutic strategy for RNA virus infections [58]. Ribavirin, a nucleoside with broad antiviral activity, has attracted considerable interest, and can induce lethal mutagenesis of Hantaan virus and poliovirus [42], [43], [59]. While ribavirin is used clinically for the treatment of both respiratory syncytial virus and hepatitis C virus, it has pleiotropic effects, and its mechanism of action in these infections is unclear [60]. Another mutagen, 5-fluorouracil, is licensed as a chemotherapeutic agent, and its antiviral activity against LCMV in animal models may predict efficacy for other arenaviruses, such as Lassa fever [61], [62]. Spurred by these results, Loeb and colleagues have identified a number of nucleoside analogs capable of inducing the lethal mutagenesis of HIV [63]–[65].

While these results are encouraging, much work needs to be done before lethal mutagenesis can be considered as a quasispecies-inspired therapeutic strategy. Some have argued for a distinction between lethal mutagenesis and error catastrophe, and have suggested, on theoretical grounds, that the above experiments do not show a true phase transition with loss of a master sequence. Indeed, careful studies of lethal mutagenesis by Lowenstein and colleagues have shown an imperfect correlation between mutational load and population extinction [46]. Pre-extinction populations exhibit marked heterogeneity in both the location and number of mutations per genome. The dynamics of extinction are further complicated by the observation that highly mutagenized genomes can accelerate extinction by interfering with the replication of their less mutated brethren [47], [66]. In that case, a mutagenized population would collapse without the genetic melting of an error catastrophe. While these discrepancies may reflect the gap between mathematical theory and biological complexity, the distinction could have “real world” implications. Mutation is a double-edged sword, and adaptive evolution can be accelerated at high mutation rates [67]. Absent a true mutational meltdown, a large pre-extinction population could serve as a reservoir for novel mutants that mediate antiviral resistance or immune escape [68]–[70].

The potential for mutagen resistance is another important concern. In principle, a virus could evolve biochemical resistance to nucleosides either by excluding the drug from its active site or by lowering its intrinsic error rate (see below). Quasispecies theory suggests that viruses could also achieve resistance by moving to flatter regions of the fitness landscape, where the density of neutral mutations is higher. Combination therapy with mutagens and traditional antivirals may minimize the probability of mutagenic escape, and work in the FMDV system is encouraging [71], [72]. The safety of nucleoside analogs is a major concern that is largely unexplored. Drug concentrations in the above studies were often in the millimolar range, and mutagenic nucleosides with a wider therapeutic index are clearly needed. Given the potential for off-target effects on host cell polymerases, therapy would likely need to be short term—a problem for HIV, where eradication of the latent reservoir is critical.

## Mutation Rate, Virulence, and Attenuation

Evolutionary theory predicts that high mutation rates are favored in a dynamic environment, and viral error rates may have been optimized by natural selection [36], [37]. For RNA viruses, low replicative fidelity generates a diverse population of variants. While these variants are generally less fit, they may quickly dominate if a sudden change in environment, such as immune pressure, shifts the corresponding fitness landscape. Conversely, a homogeneous population, generated by high replicative fidelity, would lack this flexibility and might be less successful in the dynamic host environment.

Recent work by two groups in the poliovirus system provides experimental support for this model. Drawing on experience with ribavirin and lethal mutagenesis, they hypothesized that a mutant with a low mutation rate would be less sensitive to lethal mutagenesis and resistant to ribavirin. Both groups sought to isolate ribavirin resistant mutants from a poliovirus quasispecies and recovered a variant with a single amino acid substitution in the viral polymerase (3Dpol^G64S^) [73], [74]. This mutant was relatively resistant to lethal mutagenesis, and assays for selectable markers indicated that the G64S population had a lower mutation rate and exhibited less genetic diversity (Figure 3B) [73], [75], [76]. While wild-type and G64S quasispecies replicated with similar kinetics, the former was more “fit” in direct competition assays [75], [76]. Together, these data suggested that high mutation rates confer an evolutionary advantage to RNA viruses.

**Figure 4:**
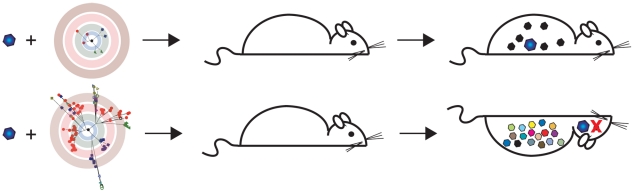
Population diversity is a virulence determinant. Results of experiments described in Vignuzzi et al. [76]. A neurovirulent clone of poliovirus was isolated from the brains of mice that had been infected with a wild-type strain. Naive mice were then reinfected with this clone as part of either a genetically constrained (top) or diverse population (bottom). Although all mice received the neurovirulent clone, only those infected with a diverse quasispecies developed disease. Subpopulations within the diverse quasispecies cooperated with the neurovirulent clone to facilitate its entry into the CNS.

The G64S quasispecies was markedly attenuated in a transgenic mouse model for poliovirus infection; the high fidelity variants were less successful at accessing the central nervous system (CNS), the site of disease [76]. Importantly, virulence was determined in large part by the diversity of the coinfecting population (Figure 4). When brain-derived polioviruses were introduced as members of the genetically homogeneous G64S population, they were not able to invade the CNS. In contrast, brain-derived viruses readily accessed the CNS when the population inoculum contained a wild-type distribution of variants. These results suggest that quasispecies diversity, rather than the selection of individual variants, correlates with enhanced virulence [76]. The demonstration of cooperative interactions among mutants in a population established the relevance of quasispecies theory to studies of viral pathogenesis. Vignuzzi and colleagues extended this model and hypothesized that the observed attenuation of the high fidelity variants could be exploited for vaccine design [77]. Focusing on the G64 position, they engineered several high fidelity mutants that were markedly attenuated in mice. These viruses stimulated high titers of neutralizing antibody in immunized mice, and provided lasting protection against subsequent lethal challenge with wild-type virus.

This work shows how quasispecies theory and its predictions can lead to the rational development of live, attenuated vaccines. Because variants with low mutation rates would be less likely to revert to wild type in a vaccinee, attenuation should be a stable phenotype and increase the safety of candidate vaccines. Caution is warranted, however, since the error rates of the high fidelity variants are still orders of magnitude greater than those of DNA viruses. It is also unclear whether reducing mutation rate will similarly affect the adaptability and virulence of other RNA viruses. Further work in other systems is clearly needed before this work can be translated into new vaccines [78], [79]. As in the case of lethal mutagenesis, the modulation of fidelity offers an opportunity to use mutation rate as a therapeutic strategy for the control of viral disease.

## Perspectives on the Future

Quasispecies theory has had a profound influence on virology, and experiments with model RNA viruses have validated many of its predictions. Considerably less is known about how its population-based models apply to the evolutionary behavior of RNA viruses in infected hosts, and it will be challenging to translate them to the complex reality of viral infection in patients. The initial studies pose as many questions as they answer. What is the best measure of viral fitness in a dynamic population? How can we use a fitness landscape model to understand selection pressure in vivo, where many such landscapes coexist? Do the principles of mutational robustness and survival of the flattest determine the behavior of populations in the host ecosystem? How does population diversity influence pathogenesis? What are the mechanisms by which variants or subpopulations cooperate, and does this have implications for coinfection?

While early forays into complex experimental systems have provided tantalizing results, much more work is needed in this area if quasispecies theory is to be translated from bench to bedside. Improved assays for characterizing viral populations are critical, and the advent of ultra high throughput, or “deep” sequencing, is particularly exciting. Several groups have used this technology to explore the complexity of mutant spectra in clinical samples from HIV- and HBV-infected patients, although the sequencing error rate presents certain limitations for studies of RNA as opposed to DNA viruses [80]–[82]. Discerning quasispecies structure from the wealth of sequencing data presents significant computational challenges as well, since current techniques are underdeveloped and do not permit us to determine which mutations are linked on the same genome. Complementary approaches, such as molecular barcoding, will also be required if we are to understand how the mutant spectrum changes temporally or spatially within an infected host [83]. Finally, future drug and vaccine studies will need to be carried out in well-defined animal models, as subtle differences can have a significant impact on experimental outcome. Despite these obstacles, we are confident that quasispecies theory will soon move out of the laboratory and begin to influence the control and treatment of viral disease.
